# Training and competence perception differences in otolaryngology and head and neck surgery training program – an anonymous electronic national survey

**DOI:** 10.1186/s12913-023-10195-2

**Published:** 2023-11-11

**Authors:** Nir Hirshoren, Tali Landau Zemer, Michal Shauly-Aharonov, Jeffrey M. Weinberger, Ron Eliashar

**Affiliations:** 1 Department of Otolaryngology / Head & Neck Surgery, Faculty of Medicine, Hadassah Hebrew-University Medical Center, Jerusalem, Israel; 2 School of Public Health and Community Medicine, Hebrew University, Jerusalem, Israel; 3 Department of Industrial Engineering and Management, Jerusalem College of Technology, Jerusalem, Israel

**Keywords:** Otorhinolaryngology Head and Neck Surgery, Training program, Residency program, Curriculum, Competence

## Abstract

**Background:**

Otorhinolaryngology / Head and Neck Surgery consists of different sub-specialties, each comprising unique characteristics and challenges. Herein, we investigate the use of a uniform national electronic questionnaire for curriculum planning. Main outcome measures: (1) Analyze the residents’ perception of the different sub-specialties training programs and their competence capabilities. (2) Identify sub-specialties requiring attention. (3) Investigate the characteristics associated with competence perception.

**Methods:**

This is a national cross sectional study. An anonymous electronic questionnaire was emailed to all registered Otorhinolaryngology / Head and Neck Surgery residents.

**Results:**

63.5% registered residents responded to the questionnaire. Two sub-specialties, Rhinology and Laryngology, are located in the extremities of the residents’ perceptions of competence and training (p < 0.0001), despite similar complexity perception (means 6.10 and 6.01, respectively). Rhinology is perceived as the most well-trained sub-specialty, both surgically and clinically (means 7.08 and 7.66, respectively), whereas Laryngology is bottom scaled (means 5.16 and 6.14, respectively). The same is true for perceived competence, surgical and clinical, in Rhinology (means 6.80 and 8.02, respectively) compared to Laryngology (means 5.04 and 6.75, respectively). Significant positive correlations were found between training, competence perception and workload (“golden training triangle”).

**Conclusions:**

Each ORL-HNS sub-specialty comprises different characteristics and a different learning curve, necessitating a tailored training program. Recognizing its sub-specialties distinctive features may assist in establishment of better-adapted learning curves in residency programs. Herein, we examine the use of anonymous electronic national survey. Laryngology, bottom ranked, is a prototype of a relatively new surgical discipline. Rhinology, ranked top by the residents, is an exemplar of a sub-specialty with an optimal ‘educational environment’. Moreover, we have established golden training triangle, implicating, highlights the essential role of institutional and senior staff for proper residency teaching. We demonstrate and advocate the benefit of using an anonymous electronic questionnaire.

**Supplementary Information:**

The online version contains supplementary material available at 10.1186/s12913-023-10195-2.

## Background

The ongoing evidence-based era, together with technology advances, intensify the requirement of adequate, repeated, evaluation of the diverse clinical and surgical skills to be achieved by surgeons. A high-quality residency training program is a key factor for ensuring a better health system with high quality health providers [1, 2]. Herein, we present the potential benefits of a national survey, conducted among Otorhinolaryngology / Head and Neck Surgery (ORL-HNS) residents for a better curriculum adaptations.

Over the decades, continuous diagnostic and therapeutic progress resulted in the establishment of several sub-specialties in ORL-HNS, each differently characterized by unique goals and challenges. The pediatric subspecialty was developed in North America in the 1960s [3]. The invention of the optic endoscopic system [4] resulted in the development of minimally invasive sino-nasal surgery in the early 1970’s [5, 6]. The recognition and understanding of the fundamental basics of voice were introduced in the early 1980’s, [7] resulting in the field of Laryngology. This professionalization led to considerable structural changes in the daily work of otorhinolaryngologists, followed by necessary adaptations in residency training programs.

The five main ORL-HNS sub-specialties in Israel are Otology, Head and Neck Surgery, Pediatric ENT, Rhinology, and Laryngology. To achieve maximal qualifications, essentially measured by clinical and surgical competence, a thorough investigation of the above five main different residency streams is compulsory. Revealing residents’ perceptions of competence in one or more of the sub-specialties may assist in establishment of better-adjusted learning curves to be incorporated into the residency programs.

## Methods

### Study aim and primary end points

The study investigate the utility of anonymous electronic national survey among surgical residents for better curriculum and training program planning.

Primary endpoints: (1) To analyze the residents’ comprehensive perception of competence in five different main surgical sub-specialties training programs. (2) To identify training gaps and sub-specialties requiring extra attention. (3) To investigate, for each sub-specialty, the training characteristics associated with the perception of competence.

### Study design and setting, ethical considerations

An anonymous electronic questionnaire was emailed to all registered ORL-HNS residents. The first question was having the participants’ written agreement (‘YES’) in order to proceed and answer the rest of the questionnaire. The study was approved and conducted according to the local Internal Review Board guidelines (Helsinki Committee. 0621-20- HMO), Hadassah medical center. Participants’ names and affiliations were not requested in order to ensure frank, truthful answers, and to protect residents refusing to participate in the study. Participants had to approve their participation (this was a digital informed consent obtained from all participants). The participants were aware of the study’s purpose. The investigation report is according to the STROBE guidelines.

In each question, the participant graded his/her perception from 1 to 10, where grades 1–2 reflected ‘very low’, 3–4 for ‘low’, 5–6 for ‘average’, 7–8 for ‘high’, and 9–10 for ‘very high’. The survey was established by national clinical educators and educational experts and by a leading member of the scientific council of the national medical association and supreme examination committee.

### Participants

All registered ORL-HNS residents, postgraduate years (PGY) 1 to 6. We categorized the participants into three seniority levels: PGY1-2, PGY3-4 and PGY5-6. We divided the training and competence perceptions into ‘surgical’ and ‘clinical’. While ‘surgical’ stands for technical considerations and operating skills, ‘clinical’ means knowledge necessary for diagnosis, investigation (e.g. laboratory, imaging, and pathology), non-surgical treatment, and follow-up. ‘Workload’ stands for case-volume, operative time, and pre / post-operative activities.

### Potential biases diminishment

A potential bias is the relatively small number of responders, which correlates to the country size. The participant percentage (63.5%) among all residents is definitely high, allowing statistical analysis and deduction. This is a National study, requiring local adjustments when implied elsewhere. Yet, the rationale of the study and its implications suit all training programs and countries.

### Statistical analysis

Statistical analyses were performed using R software, version 3.5.1 and Microsoft Excel. Basic participants’ perceptions were summarized using means, medians, and inter-quartile ranges when appropriate. Repeated measures ANOVA, both parametric (F-test) and non-parametric (Friedman test), were conducted in order to test the hypothesis that there were significant differences between mean scores in the five sub-specialties. In case of evidence that the means were not the same, post-hoc tests (i.e. paired t-test and Wilcoxon sign-rank test) were performed in order to identify which sub-specialties were different. Results were adjusted by Holm’s correction for multiple comparisons. Levene’s test was performed to decide if there were significant differences between the variances in the five sub-specialties. In addition, Pearson correlations between the different characteristics of each sub-specialty were calculated and their p-values were corrected for multiple comparisons using Holm’s correction. P-value of 0.05 or less was considered statistically significant.

## Results

Overall, 92 residents out of 145 registered ORL-HNS residents (63.5%) participated in our study. Of these, 24 were of the youngest (PGY1-2) group, 33 were PGY3-4 and 35 were senior residents, PGY5-6. One third of the participants (n = 32) have already taken at least part of the required residency qualifying exams.

Otology was perceived as the most complex sub-specialty (mean 7.9). Rhinology, Laryngology and Pediatric ENT were perceived as less complicated, with similar complexity (means 6.10, 6.01 and 5.74, respectively). (Table 1)

**Table 1:**
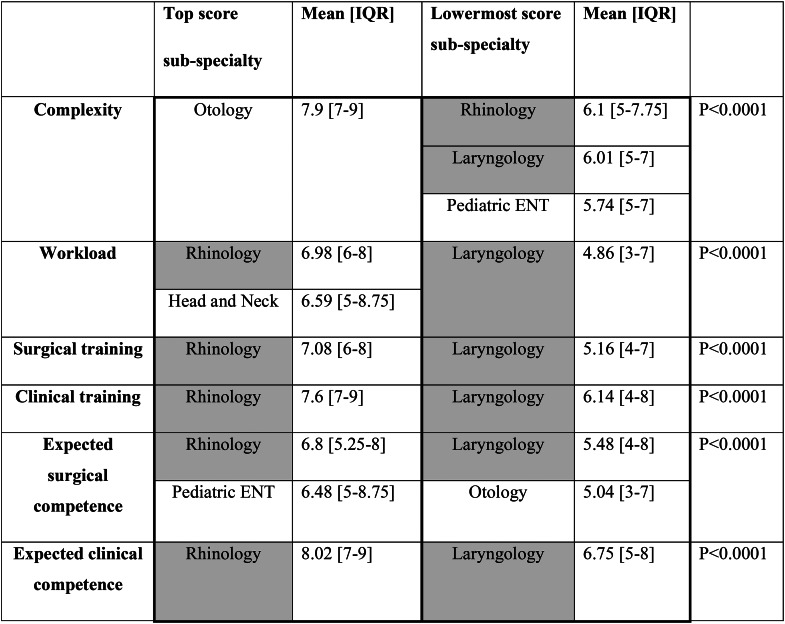
A comparison of surgical sub-specialties’ *highest* and *lowermost* scores (range 1–10) ENT: Ear Nose and Throat. IQR: Interquartile range Gray color for Laryngology and Rhinology, emphasizing the two sub-specialties most often graded on the extremities

Rhinology and Head and Neck Surgery had the highest workload (mean 6.98 and 6.59, respectively), while Laryngology is perceived as the least loaded (mean 4.86).

With regard to surgical training, Rhinology was the most well-trained sub-specialty (mean 7.08), followed by Head and Neck Surgery, Otology and Pediatric ENT (mean 6.24, 6.05 and 5.92, respectively), and lastly, by Laryngology (mean 5.16) (shown in Table 1). Similarly, Laryngology had the lowest clinical training score (mean 6.14); all other sub-specialties were similar in terms of clinical training: Rhinology (mean 7.66), Head and Neck Surgery (mean 7.36), Otology (mean 7.30), and Pediatric ENT (mean 7.07).

Rhinology and Pediatric ENT had higher expected surgical competence levels (mean 6.80, 6.48, respectively), whereas in Laryngology, the residents rated their surgical competence as significantly (p < 0.0001) weaker (5.04). In terms of clinical competence, the highest was found in Rhinology (mean 8.02) and the lowest is in Laryngology (mean 6.75).

Full description of residents’ perception is depicted in supplementary Figs. 1 and 2.

No significant differences are found between the different seniority levels (PGY1-2, PGY3-4, PGY5-6) regarding complexity, workload, training and perceptions of competence.

**Fig. 1:**
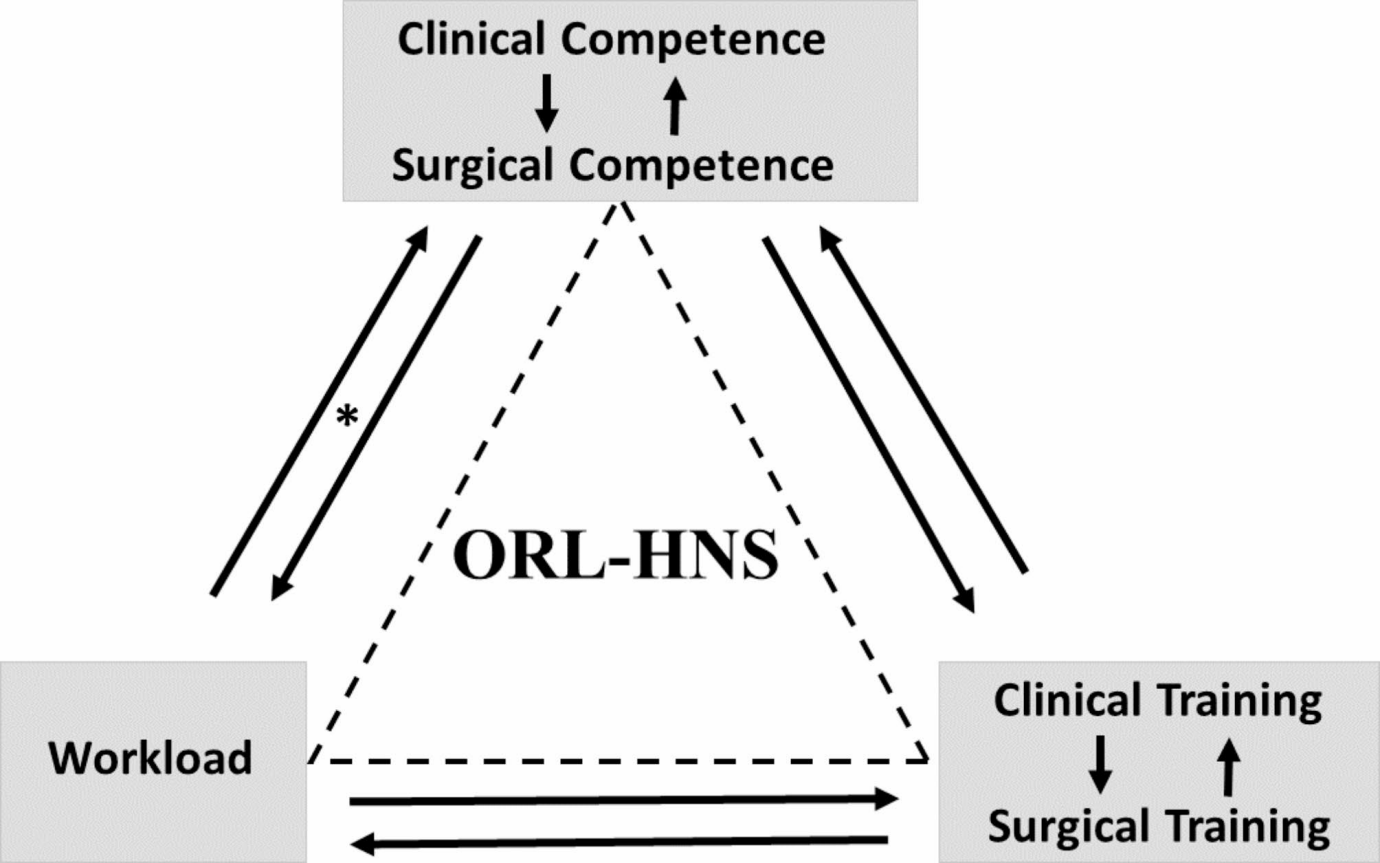
The associations between the main residents’ perceptions in the main sub-specialties; Otology, Head and Neck Surgery, Pediatric ENT, Rhinology and Laryngology

Significant positive associations were found in all sub-specialties between clinical and surgical training, clinical and surgical perception of competence, workload and training (surgical training in particular). All sub-specialties but one (Rhinology, p = 0.181) demonstrated significant associations between workload and perception of competence (both clinical and surgical). (shown in Fig. 1)

## Discussion

In this comprehensive national investigation, we have divided the surgical residency-training program into its five main streams. Each sub-specialty comprises different characteristics including workload, pre-operative / operative / post-operative intricacy, hands-on and surgical tasks, and risk for complications. Altogether, each sub-specialty has a different learning curve, necessitating a tailored training program. We all acknowledge the diversity of the ORL-HNS profession, as most surgeons in academic medical centers practice a specific sub-specialty. However, the expectations for residents should be acquirement of basic competence in all the subspecialties. The residency-training program should accordingly consist of different specific goals, skills to be achieved, allotted time, proportion in exams and emphasis for each stream. Investigation of the surgical residency-training program as perceived by the residents may attribute and assist in building a conformed curriculum, referring individually to each sub-specialty.

In our questionnaire we have deliberately avoided asking on, or using the terms, ‘peripheral’ / ‘local’ / ‘rural’ hospitals, as well as the terms ‘referral’ / ‘central’ / ‘tertiary’ hospitals. In our view, using these terms in the questionnaire could have triggered bias, by leading the participants to the so-called ‘right answers’. We have therefore used ‘workload’ and ‘case-volume’ as reliable substitutes, indicating the departments’ status.

Ongoing, repeated investigation of the residency training programs is obligatory in any accredited, adjusted system, in order to achieve better and relevant outcomes [8–10]. Different aspects in the national residency programs constantly change and improve [11]. Interestingly, different countries may apply different training programs, implicating either diverse local health system requirements, or different levels of self-investigation [12]. In addition, there are discrepancies between medical centers providing specific dedicated services, because of their experience and reputation.

On the one hand, our results revealed streams with excellent training programs and skill acquisition. On the other hand, there are sub-specialties in need of improvement. We have demonstrated two sub-specialties located in the extremities of the residents’ perception. Rhinology was top listed in most questions considering workload, surgical and clinical training, and expected competence. Laryngology, on the other hand, with a similar complexity perception, was bottom listed in most enquiries (Table 1). Since perception evolves along the residency program [13] we investigated and demonstrated similar results in the different residents’ seniority levels (PGYs). We are aware of the inevitable variances between different medical centers, [14] hence expect and acknowledge heterogeneity of the replies as depicted by inequality using the Levene’s test. Yet, the training program, similar to this investigation, is national and not institutional.

Laryngology is a prototype of a relatively new surgical discipline with lower workload, smaller amount of hands-on training, and a relatively small surgical field, managed mostly by the specialist. Moreover, only a few medical centers boast the presence of trained, experienced, post-fellowship Laryngology experts. Lack of reliable simulator [15] and cadaver courses (in contrast to the Otology, Rhinology, and Head and Neck Surgery streams) may contribute to the problem. Rhinology, ranked top by the residents, is a exemplar of a sub-specialty with an optimal ‘educational environment’, exploiting the endoscopic view on high definition monitors, with or without the aid of the image guided navigation system. The residents enjoy numerous cadaver models and simulators, with more ‘hands-on’ training, and active supervision by dedicated post-fellowship experts. Surgical complications are relatively rare and are better controlled and avoided by the senior surgeons, thus enabling residents’ progress and achievement of adequate competence levels.

According to our findings, we have established a surgical residency ‘golden training triangle’ (shown in Fig. 1) connecting different training capabilities, competence perception and workload. The golden training triangle, implicating the imperative associations between training, workload and competence, highlights the essential role of institutional and senior staff for proper residency teaching. We believe that it may apply to other surgical professions and to other training programs in different countries as well, with employment of specific and necessary adjustments.

Our findings shed light on the Israeli ORL-HNS residency-training program and may draw attention to other surgical and non-surgical medical fields training programs, in different countries, as well. Relatively new disciplines, with low capacity, less hands-on practice, few recognized post dedicated fellowship national experts, and paucity of surgical training models, may all implicate the need for a tailored learning curve, with well-designed milestones, for better residency achievements expressed particularly by the perception of competence. We highly advocate the use of an anonymous electronic questionnaire among residents.

## Conclusions and policy implications

Surgical residency is a complex, challenging, heterogenic training-program, with diverse skills to be achieved. Rhinology and Laryngology are prototypes for top and bottom graded streams, highlighting the importance of the investigation of the sub-specialties’ distinctive characteristics as clearly demonstrated by using an anonymous electronic questionnaire. Recognizing each sub-specialty’s unique features may assist in the establishment of better-adapted learning curves in residency programs.

### Electronic supplementary material

Below is the link to the electronic supplementary material.

Supplementary Material 1

Supplementary Material 2

## Data Availability

The datasets used and analyzed during the current study are available from the corresponding author on reasonable request.

## List of abbreviations

ORL-HNS: Otorhinolaryngology / Head and Neck Surgery
PGY: Post Graduate Year
ENT: Ear Nose Throat
