# Supplementary figures and images for: Training and competence perception differences in otolaryngology and head and neck surgery training program – an anonymous electronic national survey
Source: BMC Health Serv Res. 2023 Nov 11;23:1239. doi: 10.1186/s12913-023-10195-2 (PMC10638777; doi:10.1186/s12913-023-10195-2)

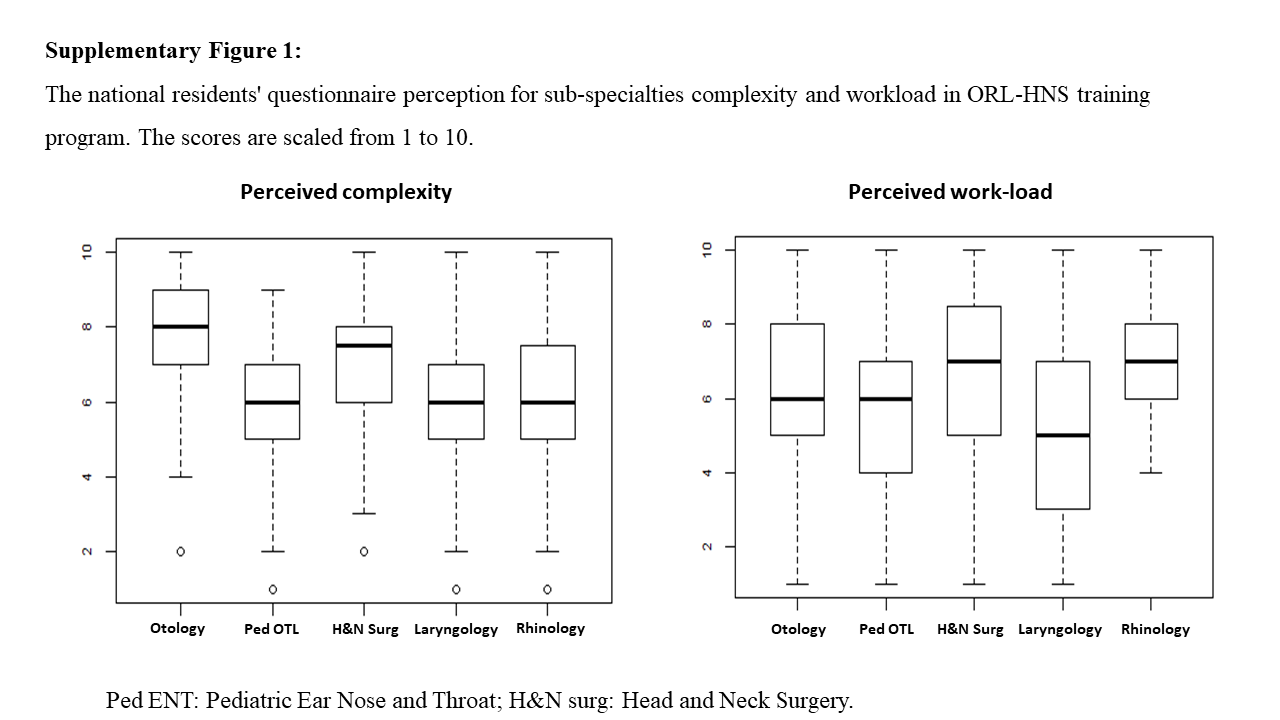

Supplement: Supplementary file 1 — Supplementary Material 1 [file 12913_2023_10195_MOESM1_ESM.tif]

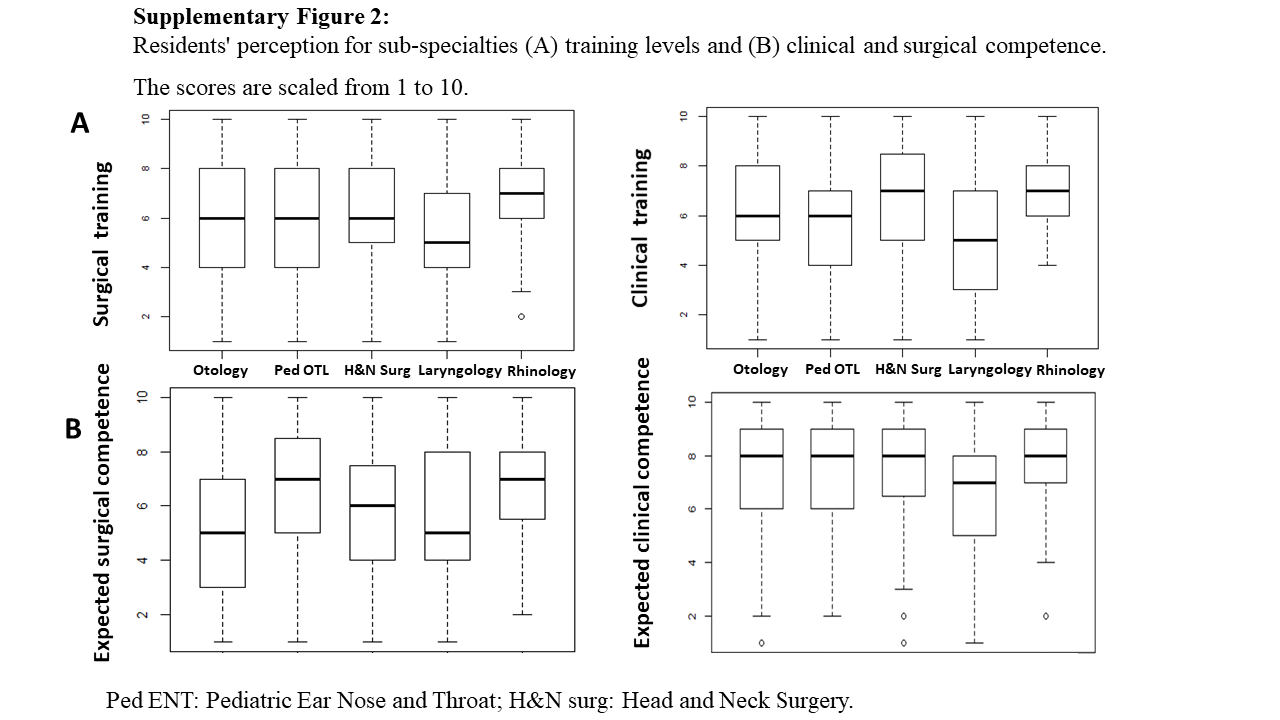

Supplement: Supplementary file 2 — Supplementary Material 2 [file 12913_2023_10195_MOESM2_ESM.tif]
